# Critical Role of Lkb1 in the Maintenance of Alveolar Macrophage Self-Renewal and Immune Homeostasis

**DOI:** 10.3389/fimmu.2021.629281

**Published:** 2021-04-22

**Authors:** Qianqian Wang, Song Chen, Tengda Li, Qiongmei Yang, Jingru Liu, Yuan Tao, Yuan Meng, Jiadi Chen, Xiaoming Feng, Zhongchao Han, Mingxia Shi, Huifang Huang, Mingzhe Han, Erlie Jiang

**Affiliations:** ^1^ State Key Laboratory of Experimental Hematology, National Clinical Research Center for Blood Diseases, Institute of Hematology & Blood Diseases Hospital, Chinese Academy of Medical Sciences & Peking Union Medical College, Tianjin, China; ^2^ Department of Hematology, The First Affiliated Hospital of Kunming Medical University, Hematology Research Center of Yunnan Province, Kunming, China; ^3^ Central Laboratory, Fujian Medical University Union Hospital, Fuzhou, China

**Keywords:** alveolar macrophages, Lkb1, self-renewal, immune function, immune homeostasis

## Abstract

Alveolar macrophages (AMs) are pivotal for maintaining lung immune homeostasis. We demonstrated that deletion of liver kinase b1 (Lkb1) in CD11c^+^ cells led to greatly reduced AM abundance in the lung due to the impaired self-renewal of AMs but not the impeded pre-AM differentiation. Mice with Lkb1-deficient AMs exhibited deteriorated diseases during airway *Staphylococcus aureus* (*S*. *aureus*) infection and allergic inflammation, with excessive accumulation of neutrophils and more severe lung pathology. Drug-mediated AM depletion experiments in wild type mice indicated a cause for AM reduction in aggravated diseases in Lkb1 conditional knockout mice. Transcriptomic sequencing also revealed that Lkb1 inhibited proinflammatory pathways, including IL-17 signaling and neutrophil migration, which might also contribute to the protective function of Lkb1 in AMs. We thus identified Lkb1 as a pivotal regulator that maintains the self-renewal and immune function of AMs.

## Introduction

Alveolar macrophages (AMs) act as the first sentinels of the pulmonary innate immune system (1), whose niche in the alveolar space is important for monitoring pulmonary homeostasis. Under steady conditions, AMs are primarily derived from fetal monocytes and maintain their niche by proliferative self-renewal (1, 2). Studies have shown that AM development is highly dependent on granulocyte macrophage colony-stimulating factor (GM-CSF) receptor signaling, as both *Csf2ra-* and *Csf2rb*-deficient mice are devoid of AMs (3). GM-CSF has a lung-specific role in the perinatal development of AMs *via* induction of PPAR-γ in fetal monocytes, which may promote the differentiation of pre-AMs into mature AMs (4). In addition, reports have identified that another cytokine, transforming growth factor-β (TGF-β), is critical for the development and maturation of AMs (2, 5). Although these studies have examined the development of AMs, the potential molecular mechanisms underlying the self-renewal of terminally differentiated AMs are not well understood.

The number of AMs is maintained at constant levels to sustain pulmonary homeostasis and respiratory function and to initiate the response to inhaled pathogens in the lung (1). AMs have a role in regulating response to infections and epithelial injury. AMs can interact with alveolar epithelial cells, dendritic cells (DCs) and T cells through cell surface receptors and chemokine/cytokine networks to finely regulate the immune response to environmental pathogens and lung particles (6, 7). Anti-inflammatory molecules are highly expressed in AMs, in which the CD200 receptor can bind its ligand on alveolar epithelial cells to inhibit the inflammatory response induced by the Toll-like receptor (8). In addition, signal-regulatory protein-α, which is expressed on AMs, inhibits macrophage activation and phagocytosis by binding surfactant proteins A and D (6, 9). AMs also release TGF-β, which prevents DC-mediated activation of effector T cells to inhibit immune responses (1). AMs recognize opsonized microorganisms and facilitate pathogen clearance using different receptors, such as immunoglobulin receptors and complement receptors (10, 11). How AMs recognize the presence of pathogens or products of injury and respond directly to provide optimal host protection and the potential molecules that regulate these responses remain to be discovered.

Liver kinase b1 (Lkb1), a serine–threonine kinase and tumor suppressor, was first identified in Peutz–Jeghers syndrome (12, 13). Lkb1 participates in multiple processes, including cell polarity, cell cycle arrest, embryonic development, hematopoietic stem cell maintenance, apoptosis and metabolism (14). In various tissues, Lkb1 plays an important role in regulating glucose homeostasis and energy metabolism. In addition, there is also evidence that has highlighted a prominent role for Lkb1 in the function of macrophages (15). Lkb1 inhibits the activation and inflammatory function of innate macrophages (16, 17). However, the roles of Lkb1 in regulating the homeostasis of AMs and specific airway inflammation need to be investigated.

Here, we investigated the role of Lkb1 in the maintenance of AM self-renewal and immune homeostasis. We found that disruption of Lkb1 impaired the self-renewal of AMs, contributing to the reduction of AMs, which led to aggravated symptoms and excessive accumulation of neutrophils in *Staphylococcus aureus* (*S. aureus*) pneumonia and asthma. In addition, we found that many critical immune genes of AMs are regulated by Lkb1. Transcriptomic sequencing also revealed that Lkb1 inhibits proinflammatory pathways, including IL-17 signaling and neutrophil migration pathways. Therefore, we identified Lkb1 as a crucial maintainer of AM self-renewal and immune homeostasis.

## Materials and Methods

### Mice

Mice were housed in specific pathogen-free barrier facilities. Mice were used in accordance with protocols approved by the Institutional Animal Care and Use Committee at the Institute of Hematology, Chinese Academy of Medical Sciences. C57BL/6 mice, *Lkb1* ^f/f^ mice, *AMPKα1* ^f/f^ mice and *Cd11c* ^Cre^ mice were all purchased from Jackson Laboratories (Bar Harbor, ME, USA). The mice were backcrossed with C57BL/six mice for at least seven generations. *Lkb1* ^f/f^ mice and *AMPKα1* ^f/f^ mice were crossed with *Cd11c* ^Cre^ mice to generate *Cd11c* ^Cre^ *Lkb1* ^f/f^ mice and *Cd11c* ^Cre^ *AMPKα1* ^f/f^ mice, respectively. Mice were used at 6–8 weeks old, unless otherwise indicated.

### Cell Isolation and Preparation

For analysis of cell surface markers, single cell suspensions were prepared from BAL fluid, lung, medLN, kidney, liver and thymus. To obtain BAL fluid cells, mice were sacrificed and pinned upright to a dissection board. The tracheas were exposed, and a small opening was cut with surgical scissors. A 1 ml syringe with a needle was inserted into the tracheal opening, and the alveolar cavity was washed with 500 µl PBS at least six times. BAL fluid was obtained and transferred to a 10 ml tube. The lung, medLN, kidney, liver and thymus single-cell suspensions were prepared using the same procedure as follows. The target tissues were finely ground with a filter, and the red blood cells were lysed with 200 µl erythrocyte lysate (Solarbio) for 10 min at 4°C. Leukocytes were washed with PBS to obtain single cell suspensions.

### Flow Cytometry

Flow cytometry antibodies and their clone numbers are listed in **Table S2**. Cell surface staining was performed at 4°C for 30–40 min in the dark. The cells were stimulated with phorbol myristate acetate (50 ng/ml) and ionomycin (500 ng/ml) for 4–5 h for intracellular cytokine staining. Intracellular staining with Ki67 was performed using Foxp3 staining kits (eBioscience). The antibodies we used were purchased from Biolegend, BD Bioscience, eBioscience, and Invitrogen. The indicated AM populations were sorted by FACSAria III (BD Biosciences) from BAL fluid or lung. The purity of sorted populations was >99%, unless otherwise indicated. Data were obtained on a FACSCanto II (BD Biosciences) and analyzed using FlowJo software (FlowJo LLC, Ashland, Oregon).

### Bone Marrow Chimera Model

Recipient CD45.1^+^CD45.2^+^ mice (8–10 weeks old) were lethally irradiated twice with 400 cGy. Irradiated recipient mice were then intravenously injected with 1 × 10^7^ BM cells, which were from *Lkb1* ^f/f^ (CD45.1^+^) mice and *Cd11c* ^Cre^ *Lkb1* ^f/f^ (CD45.2^+^) mice at a ratio of 3:1. Chimeric mice were housed in sterile caging for 8 weeks to allow for reconstitution.

### Apoptosis Detection

According to the instructions of the manufacturers, Annexin V and PI staining were performed with an apoptosis detection kit (Biolegend) to test the apoptosis of AMs from *Lkb1* ^f/f^ mice and *Cd11c* ^Cre^ *Lkb1* ^f/f^ mice by flow cytometry (Canto II, BD Biosciences).

### *S. aureus* Pneumonia Model

*Lkb1* ^f/f^ and *Cd11c* ^Cre^ *Lkb1* ^f/f^ mice were intranasally challenged with 5 × 10^4^ colony-forming units (CFU) per animal of *S. aureus* in 50 μl PBS. The mouse body weight was monitored until the mouse died or up to 6 days. The secretion of inflammatory cytokines by T cells in the lung and medLN and the percentage of neutrophils and eosinophils in the lung and BAL fluid were analyzed by flow cytometry 3 days after challenge. BAL fluid was obtained for culture on day 3 after challenge to calculate CFU.

### Encapsome and Clodrosome

Encapsome and Clodrosome are the products of Encapsula NanoSciences (SKU# CLD-8901). The clodronate was encapsulated with liposomes to form a multilamellar liposome suspension, which is called Clodrosome. Encapsome is the control liposome of Clodrosome, in which clodronate was not added to the liposomes. The larger particles were removed from the liposomes, and all the preparation processes were performed under sterile conditions. When treated with Clodrosome, the phagocytic cells in animals recognize liposomes as invasive foreign particles and remove liposomes by phagocytosis. Then, the liposomes released the clodronate into the cytosol, leading to cell death. The recognition and phagocytosis mechanism of Encapsome was the same as that of Clodrosome. Since the Encapsome did not contain clodronate, the phagocytic cells were not killed.

### AMs Depletion

*Lkb1* ^f/f^ mice were intranasally challenged with 100 μl (500 μg) Encapsome or Clodrosome (5 mg/ml, from Encapsula NanoSciences, SKU# CLD-8901) per mouse once a day, one to two times a week.

### HDM Asthma Model

*Lkb1* ^f/f^ and *Cd11c* ^Cre^ *Lkb1* ^f/f^ mice were intraperitoneally anesthetized with chloral hydrate (Solarbio) and then intranasally challenged with 25 μg HDM (Greer Laboratories Inc., Lenoir, NC, USA) per animal in 50 μl PBS on days 0, 2, 4, 7, 9, 11, 14, 15, and 16 (12). On day 17, the mice were sacrificed, and the secretion of inflammatory cytokines by T cells in the lung and medLNs and the percentage of neutrophils and eosinophils in the lung and BAL fluid were analyzed by flow cytometry.

### RNA-Seq

CD45^+^ CD11c^+^ SiglecF^+^ AMs in the lungs of *Lkb1* ^f/f^ and *Cd11c* ^Cre^ *Lkb1* ^f/f^ mice were sorted for RNA sequencing. The purity of sorted populations was more than 99%. RNA was extracted using TRIzol reagent (Invitrogen) for RNA sequencing analysis on the BGISEQ500 platform (BGI-Shenzhen, China). DESeq2 (v1.4.5) was used for the differential expression analysis (18) with a Q value ≤0.05. RNA transcriptome sequencing datasets were analyzed and edited using GlueGO in Cytoscape software (19). We have submitted the RNA-Seq datasets to the Gene Expression Omnibus (GEO), and the accession number is GSE167349.

### Histological Analyses

Samples were harvested from the lungs on day 3 after *S. aureus* infection and fixed with 10% formalin immediately. Samples were washed using 70% ethanol and embedded in paraffin. Then, they were cut into 6-μm thick slices and stained with hematoxylin and eosin. The presented data are from individual lung. All slices used for analysis were encoded and read blindly. Photomicrographs were taken at a 10 × and 40 × magnification.

### Lung Injury Score

Two investigators quantified the lung injury score blindly according to published criteria of American Thoracic Society Documents (20). Each sample was analyzed for at least five fields. Five independent parameters of lung injury scoring system were as follows: A. neutrophils in the alveolar space; B. neutrophils in the interstitial space; C. hyaline membranes; D. proteinaceous debris filling the airspaces; E. alveolar septal thickening. The calculation was: scores = [(20 × A) + (14 × B) + (7 × C) + (7 × D) + (2 × E)]/(number of fields × 100). The resulting score is a continuous value between zero and one (inclusive).

### Statistics

Data were analyzed using GraphPad Software (Prism 5.00, San Diego, CA, USA). The statistical significance of the difference between the two groups was calculated, and the *P*-value was determined. According to the number of comparison groups, Student’s *t*-test or two-way ANOVA was performed. *P <*0.05 was considered statistically significant. *P <0.05; **P <0.01; ***P <0.001.

## Results

### Loss of Lkb1 Leads to a Marked Reduction of AMs

**Figure 1:**
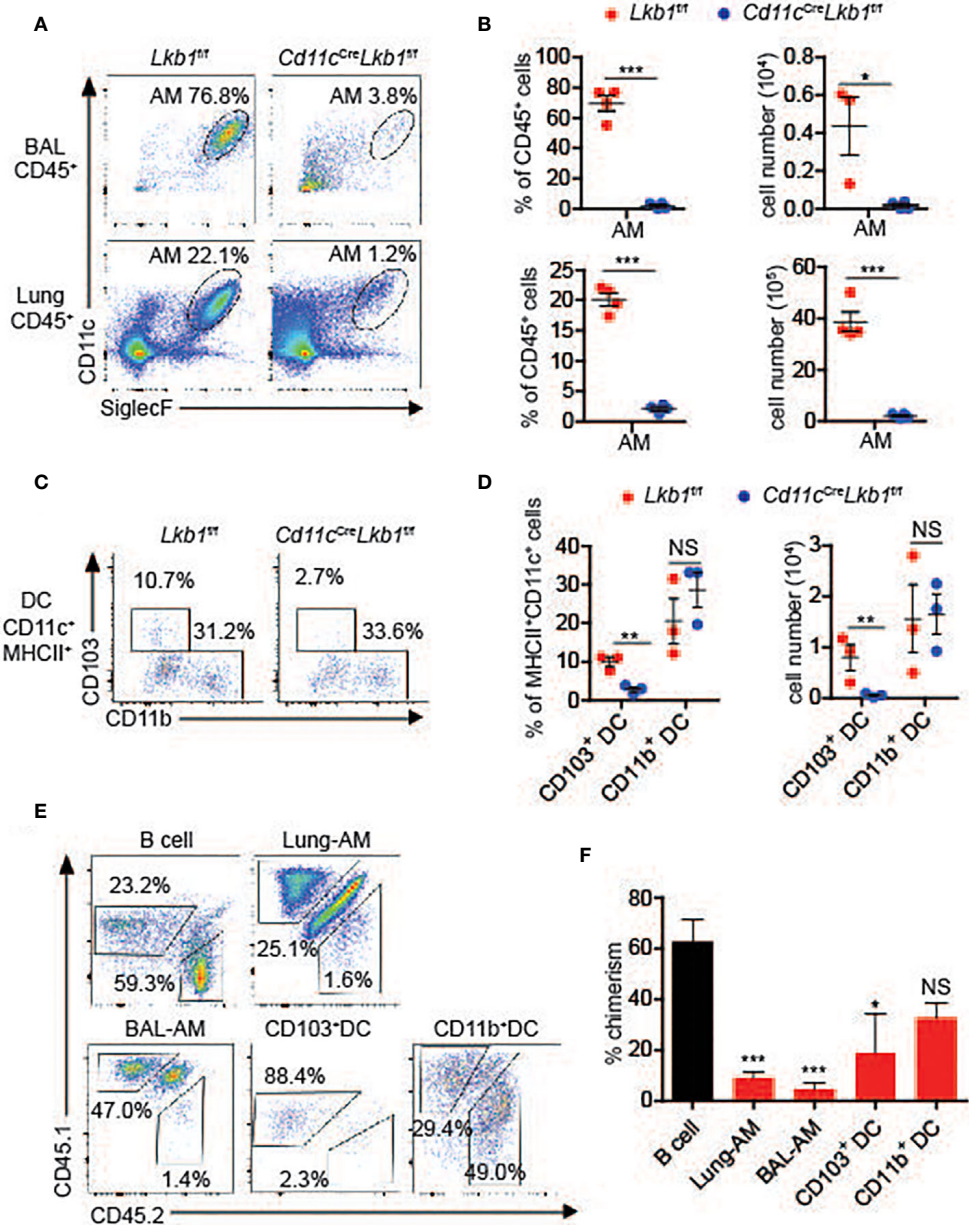
Loss of Lkb1 leads to a marked reduction in AMs in *Cd11c* ^Cre^ *Lkb1* ^f/f^ mice. **(A)** The AM frequency among CD45^+^ leucocytes in BAL fluid and lungs from *Lkb1* ^f/f^ mice and *Cd11c* ^Cre^ *Lkb1* ^f/f^ mice was analyzed by flow cytometry. **(B)** The percentages and absolute numbers of AMs in BAL fluid and lungs from *Lkb1* ^f/f^ mice and *Cd11c* ^Cre^ *Lkb1* ^f/f^ mice (n = 4). **(C)** Flow cytometry analysis of CD103^+^ and CD11b^+^ DC frequencies among CD11c^+^ MHCII^+^ DCs in the lungs of *Lkb1* ^f/f^ mice and *Cd11c* ^Cre^ *Lkb1* ^f/f^ mice. **(D)** The percentages and absolute numbers of CD103^+^ and CD11b^+^ DCs in the lungs of *Lkb1* ^f/f^ mice and *Cd11c* ^Cre^ *Lkb1* ^f/f^ mice (n = 4). **(E)** Mixed bone marrow chimeras were established by mixing Lkb1-deficient (CD45.2) BM cells and wild type (CD45.1) BM cells (3:1) and transferring them to CD45.1^+^ CD45.2^+^ host mice. Chimeras were analyzed by flow cytometry. **(F)** The bar chart shows the frequency of chimerism contributed by Lkb1-deficient cells to the indicated control B cells (n = 3–5). Each symbol **(B, D)** represents a mouse, and the results are presented as the mean ± S.D., NS (not significant), *P >* 0.05, *P < 0.05, **P < 0.01, ***P < 0.001, (by Student’s *t*-test) **(B, D, F)**. All data represent at least three independent experiments.

CD11c is highly expressed on AMs, and studies have shown that *Lysm* ^Cre^-mediated recombination results in inefficient gene deletion in AMs (1, 4, 21). Therefore, we used *Cd11c* ^Cre^ *Lkb1* ^f/f^ mice, in which Lkb1 was deleted in CD11c^+^ cells, including DCs and macrophages, to explore the function of Lkb1 in AMs. Interestingly, we found that the numbers of AMs and CD103^+^ DCs were prominently decreased in bronchoalveolar lavage (BAL) fluid and/or lungs from *Cd11c* ^Cre^ *Lkb1* ^f/f^ mice (**Figures 1A–D**). Conversely, lung interstitial macrophages (IMs), lung CD11b^+^ DCs and DCs in other tissues, including the kidney, liver and thymus, were unaffected (**Figures 1C, D** and **Figures S1A–C**). We then investigated whether cell-intrinsic or cell-extrinsic factors contributed to AM reduction using a mixed bone marrow chimera model in which Lkb1-deficient (CD45.2^+^) bone marrow (BM) cells and wild type (CD45.1^+^) BM cells were mixed at a ratio of 3:1 and then transferred into CD45.1^+^ CD45.2^+^ host mice. We observed significant impairment only in AM and CD103^+^ DC accumulation (**Figures 1E, F** **)**, indicating that the homeostatic defects of AMs and CD103^+^ DCs were cell intrinsic.

### Impaired Self-Renewal Contributes to the Reduction of Lkb1-Deficient AMs

**Figure 2:**
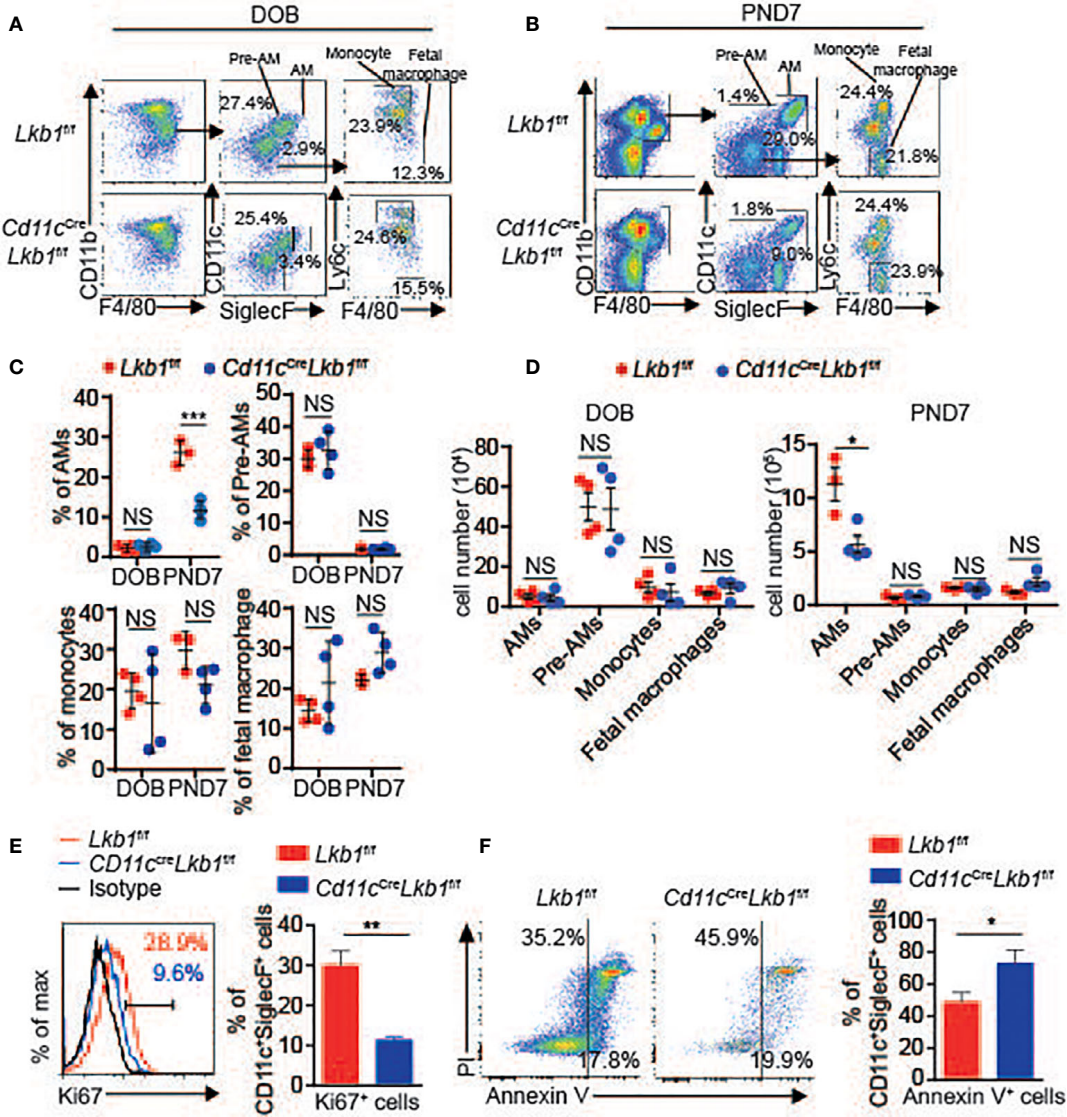
Lkb1 deletion leads to increased apoptosis and impaired proliferation. **(A, B)** Representative flow chart showing the gating scheme to identify mature AMs (gated as CD11c^+^ SiglecF^+^ F4/80^+^ CD11b^int^), pre-AMs (gated as CD11c^+^ SiglecF^−^ F4/80^+^ CD11b^int^), monocytes (gated as CD11b^high^ Ly6c^+^ CD11c^-^ SiglecF^−^ F4/80^low^) and fetal macrophages (gated as CD11b^+^ F4/80^+^ Ly6c^−^ CD11c^−^ SiglecF^-^) in the lungs of *Lkb1* ^f/f^ and *Cd11c* ^Cre^ *Lkb1* ^f/f^ mice on DOB **(A)** and PND7 **(B)**. **(C, D)** The percentages **(C)** and absolute numbers **(D)** of mature AMs, pre-AMs, monocytes and fetal macrophages in the lungs of *Lkb1* ^f/f^ and *Cd11c* ^Cre^ *Lkb1* ^f/f^ mice on DOB and PND7 (n = 3–4). **(E)** Expression of Ki67 in AMs from *Lkb1* ^f/f^ and *Cd11c* ^Cre^ *Lkb1* ^f/f^ mice (n = 3). **(F)** Annexin V and PI staining of AMs and quantification of apoptotic AMs (assessed as Annexin V-positive cells) from *Lkb1* ^f/f^ and *Cd11c* ^Cre^ *Lkb1* ^f/f^ mice (n = 3). Each symbol **(C, D)** represents a mouse, and the results are presented as the mean ± S.D., NS (not significant) *P >* 0.05, *P < 0.05, **P < 0.01, ***P < 0.001, (by Student’s *t*-test) **(C**, **D**, **E**, **F)**. All data represent at least three independent experiments.

The development of AMs, including the stepwise process from monocytes, prealveolar macrophages (pre-AMs) and mature AMs in neonatal mice, is essential for maintaining the AM population (1). To determine whether the development of AMs was retarded and participated in their absence, we analyzed the numbers of monocytes, pre-AMs and mature AMs in the lungs of *Cd11c* ^Cre^ *Lkb1* ^f/f^ and *Lkb1* ^f/f^ mice on DOB (day of birth) and PND7 (postnatal day 7). The mature AM population was comparable between *Lkb1* ^f/f^ and *Cd11c* ^Cre^ *Lkb1* ^f/f^ mice on DOB. Although the percentage and absolute number of mature AMs in *Cd11c* ^Cre^ *Lkb1* ^f/f^ mice were significantly reduced, we observed that the percentages and absolute numbers of monocytes, fetal macrophages and pre-AMs were comparable in *Lkb1* ^f/f^ and *Cd11c* ^Cre^ *Lkb1* ^f/f^ mice at PND7 (**Figures 2A–D**). These results indicated that the reduction in the AM pool occurred postnatally but not prenatally. Since the AM population is autonomously maintained by proliferative self-renewal throughout life (1), we thus examined whether self-renewal was impaired and contributed to AM reduction in *Cd11c* ^Cre^ *Lkb1* ^f/f^ mice. Results showed that Lkb1-deficient AMs exhibited increased apoptosis and reduced proliferation compared to wild type AMs (**Figures 2E, F** **)**. These results suggest that impaired self-renewal rather than AM development contribute to AM reduction in *Cd11c* ^Cre^ *Lkb1* ^f/f^ mice.

### *Cd11c* ^Cre^ *Lkb1* ^f/f^ Mice are More Susceptible to *S. aureus* Infection

**Figure 3:**
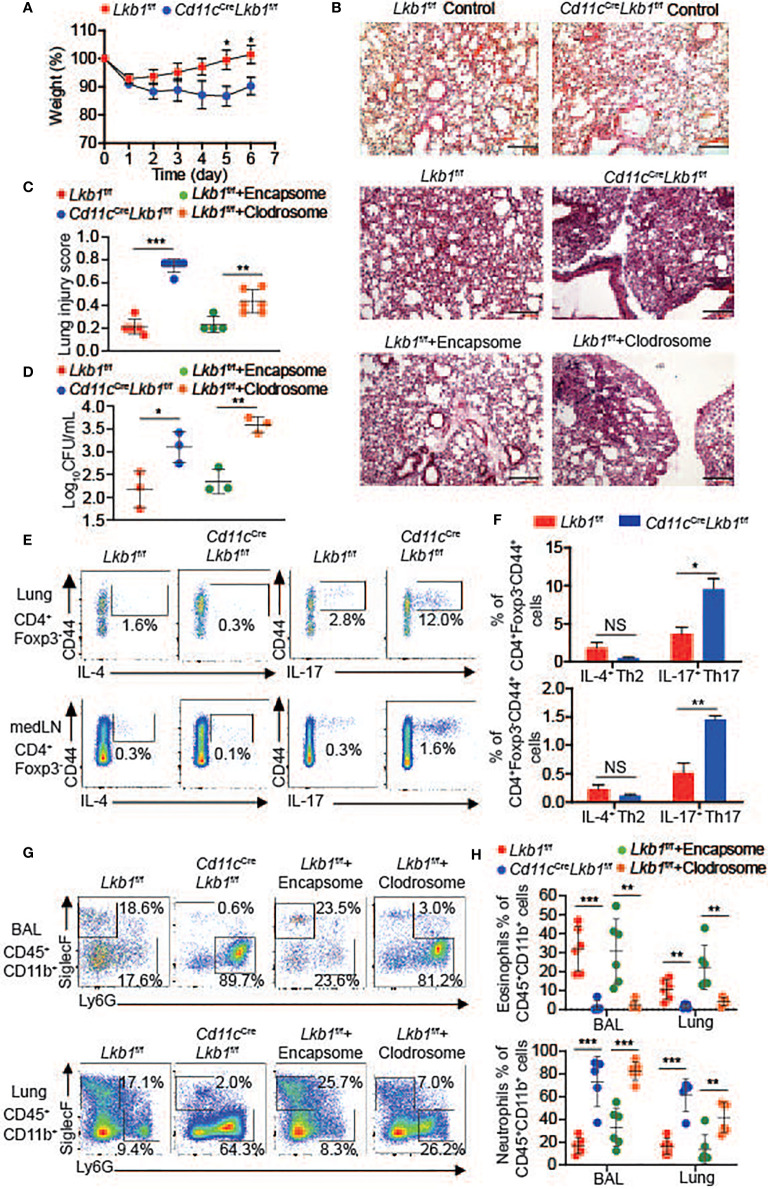
*Cd11c* ^Cre^ *Lkb1* ^f/f^ mice are more susceptible to *S. aureus* infection. **(A)** The relative weights of *Lkb1* ^f/f^ and *Cd11c* ^Cre^ *Lkb1* ^f/f^ mice challenged with *S. aureus* intranasally (n = 9–11). **(B)** Images show histopathological sections of lungs from naive *Lkb1* ^f/f^ mice, *Cd11c* ^Cre^ *Lkb1* ^f/f^ mice, and *Lkb1* ^f/f^, *Cd11c* ^Cre^ *Lkb1* ^f/f^ and *Lkb1* ^f/f^ mice intranasally administered Encapsome or Clodrosome, intranasally challenged with *S. aureus*, and stained with hematoxylin and eosin (scale bar, 100 μm). **(C)** The lung injury score was evaluated blindly by two independent investigators (n = 6). **(D)** Quantification of BAL fluid CFUs cultured for 48 h from *Lkb1* ^f/f^, *Cd11c* ^Cre^ *Lkb1* ^f/f^ and *Lkb1* ^f/f^ mice intranasally administered Clodrosome or Encapsome and intranasally challenged with *S. aureus*. **(E, F)** Flow cytometry analysis **(E)** and the frequencies **(F)** of CD4^+^ Foxp3^−^ CD44^+^ IL-4^+^ Th2 cells or CD4^+^ Foxp3^−^ CD44^+^ IL-17^+^ Th17 cells in the lung and medLNs from *Lkb1* ^f/f^ and *Cd11c* ^Cre^ *Lkb1* ^f/f^ mice challenged with *S. aureus* intranasally (n = 3). **(G, H)** Flow cytometry analysis **(G)** and quantification of the percentages **(H)** of eosinophils (CD45^+^ CD11b^+^ Siglec-F^+^) and neutrophils (CD45^+^ CD11b^+^ Ly6G^+^) in BAL fluid and lungs from *Lkb1* ^f/f^, *Cd11c* ^Cre^ *Lkb1* ^f/f^ and *Lkb1* ^f/f^ mice intranasally administered Encapsome or Clodrosome and challenged with *S. aureus* intranasally (n = 3). Each symbol **(C, D, H)** represents a mouse, and the results are presented as the mean ± S.D., NS (not significant) *P >* 0.05, *P < 0.05, **P < 0.01, ***P < 0.001 (by Student’s *t*-test) **(C, D, F, H)**, two-way ANOVA **(A)**. All data represent at least two to three independent experiments.

The AM pool is maintained at constant levels to ensure lung homeostasis, respiratory function and the pulmonary response of scavenging inhaled pathogens (22). Therefore, we detected the impact of decreased AM number on the pulmonary response using an *S. aureus* pneumonia model. Compared to *Lkb1* ^f/f^ mice, *Cd11c* ^Cre^ *Lkb1* ^f/f^ mice presented much more body weight loss and severe inflammation by pathologic analysis (**Figures 3A–C** and **Figure S2A**). Moreover, we evaluated the bacterial scavenging capacity of AMs by culturing BAL fluid from *Lkb1* ^f/f^ and *Cd11c* ^Cre^ *Lkb1* ^f/f^ mice challenged with *S. aureus* pneumonia. BAL fluid from *Cd11c* ^Cre^ *Lkb1* ^f/f^ mice produced more colonies than that from *Lkb1* ^f/f^ mice, indicating that *Cd11c* ^Cre^ *Lkb1* ^f/f^ mice had defects in bacterial scavenging capacity (**Figure 3D**). In addition, we observed an increased percentage of T helper 17 (Th17) cells in the lung and mediastinal lymph node (medLN) in *Cd11c* ^Cre^ *Lkb1* ^f/f^ mice (**Figures 3E, F** **)**. There is evidence that IL-17 enhances neutrophil accumulation under inflammatory conditions (23, 24). Indeed, an increased frequency of neutrophils and a significant reduction in eosinophils were observed in the lungs and BAL in *Cd11c* ^Cre^ *Lkb1* ^f/f^ mice (**Figures 3G, H** **)**. These results indicate that Th17/neutrophilic inflammation was driven by Lkb1 ablation in AMs. We next determined whether loss of AMs also contributes to neutrophilic lung inflammation during *S. aureus* pneumonia. *Lkb1* ^f/f^ mice intranasally administered Clodrosome but not control liposomes-Encapsome exhibited approximately 70% depletion of AMs with no change in DCs, including CD11b^+^ DCs or CD103^+^ DCs (**Figures S3A–D**). Consistent with the phenotype in *Cd11c* ^Cre^ *Lkb1* ^f/f^ mice, *Lkb1* ^f/f^ mice without AMs also exhibited more severe pathology, impaired bacteria-scavenging capacity and an imbalance between eosinophils and neutrophils in the lung during *S. aureus* pneumonia (**Figures 3B–D, G, H** and **Figure S2A**). Although the potential role of CD103^+^ DCs may not be completely excluded, AM deletion in *Lkb1* ^f/f^ mice caused almost the same degree of changes in lung pathology and neutrophil and eosinophil variation as observed in *Cd11c* ^Cre^ *Lkb1* ^f/f^ mice; thus, we concluded that AMs exerted a dominant role in *S. aureus* infection and might be involved in maintaining the balance between eosinophils and neutrophils in the lung during *S. aureus* pneumonia.

### *Cd11c* ^Cre^ *Lkb1* ^f/f^ Mice Develop More Severe Pathology in Asthma

**Figure 4:**
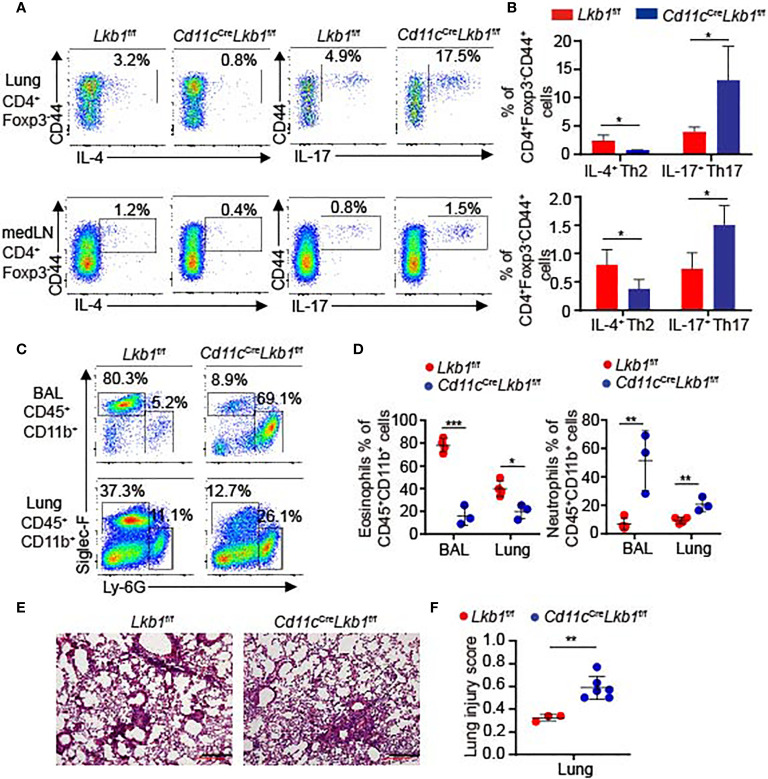
*Cd11c* ^Cre^ *Lkb1* ^f/f^ mice developed more severe pathology in asthma. **(A, B)** Flow cytometry analysis **(A)** and the frequency **(B)** of CD4^+^ Foxp3^-^ CD44^+^ IL-4^+^ Th2 cells or CD4^+^ Foxp3^-^ CD44^+^ IL-17^+^ Th17 cells in the lung and medLNs from *Lkb1* ^f/f^ and *Cd11c* ^Cre^ *Lkb1* ^f/f^ mice intranasally challenged with HDM allergens (n = 3–4). **(C)** Flow cytometry analysis of eosinophils (CD45^+^ CD11b^+^ Siglec-F^+^) and neutrophils (CD45^+^ CD11b^+^ Ly6G^+^) in BAL fluid and lungs from *Lkb1* ^f/f^ and *Cd11c* ^Cre^ *Lkb1* ^f/f^ mice intranasally challenged with HDM allergen. **(D)** Quantification of the percentages of eosinophils and neutrophils in BAL fluid and lungs from *Lkb1* ^f/f^ and *Cd11c* ^Cre^ *Lkb1* ^f/f^ mice intranasally challenged with HDM allergen (n = 3–4). **(E)** Images show histopathological sections of lungs from *Lkb1* ^f/f^ and *Cd11c* ^Cre^ *Lkb1* ^f/f^ mice intranasally challenged with HDM allergen and stained with hematoxylin and eosin (scale bar, 100 μm). **(F)** The lung injury score was evaluated blindly by two independent investigators (n = 3–6). Each symbol **(D, F)** represents a mouse, and results are presented as the mean ± S.D., *P < 0.05, **P < 0.01, ***P < 0.001, (by Student’s *t*-test) **(B, D, F)**. All data represent at least three independent experiments.

We also utilized an asthma model to investigate whether the lung inflammatory response was functionally affected in *Cd11c* ^Cre^ *Lkb1* ^f/f^ mice. *Lkb1* ^f/f^ and *Cd11c* ^Cre^ *Lkb1* ^f/f^ mice were challenged with house dust mite (HDM) allergen intranasally. We observed a significant reduction in IL-4-producing T helper 2 (Th2) cells and an increased frequency of IL-17-producing Th17 cells in the lungs and medLNs of *Cd11c* ^Cre^ *Lkb1* ^f/f^ mice (**Figures 4A, B** **)**. The typical feature of Th2-polarized allergies is the robust recruitment of eosinophils, whereas IL-17 increases neutrophil recruitment (25). Remarkably, we observed that eosinophil accumulation was decreased, while the percentages of neutrophils were significantly increased in BAL fluid and lungs from *Cd11c* ^Cre^ *Lkb1* ^f/f^ mice (**Figures 4C, D** **)**. Moreover, *Cd11c* ^Cre^ *Lkb1* ^f/f^ mice manifested more severe pathology in the lung than *Lkb1* ^f/f^ mice (**Figures 4E, F** **)**. These results indicate that the immunoprotective function is impaired in *Cd11c* ^Cre^ *Lkb1* ^f/f^ mice during allergic inflammation. AMs were directly deleted by intranasal administration of Clodrosome in *Lkb1* ^f/f^ mice to verify the crucial role of AMs in asthma. Although *Lkb1* ^f/f^ mice with AM depletion developed more severe pathology and displayed defects in recruiting eosinophils in the lung (**Figures S4A–D**), no significant increase in neutrophils was observed in the lungs of *Lkb1* ^f/f^ mice in the absence of AMs. These results suggest that AMs exert a dominant role in protecting the host from allergic inflammation and participate in regulating the balance of neutrophils and eosinophils during lung inflammation.

### Lkb1 Regulates Critical Immune and Metabolic Gene Expression in AMs

Adenosine monophosphate-activated protein kinase (AMPK) is an important downstream target of Lkb1 for regulating metabolism (14). To investigate whether AMPK was involved in AM reduction caused by Lkb1 deletion, we generated *Cd11c* ^Cre^ *AMPKα1* ^f/f^ mice in which AMPK was specifically deleted in CD11c^+^ cells. However, we found that the numbers of AMs or DCs were not significantly different between *AMPKα1* ^f/f^ and *Cd11c* ^Cre^ *AMPKα1* ^f/f^ mice (**Figures S5A–D**). These results indicate that the function of Lkb1 in maintaining AM abundance is independent of AMPK.

**Figure 5:**
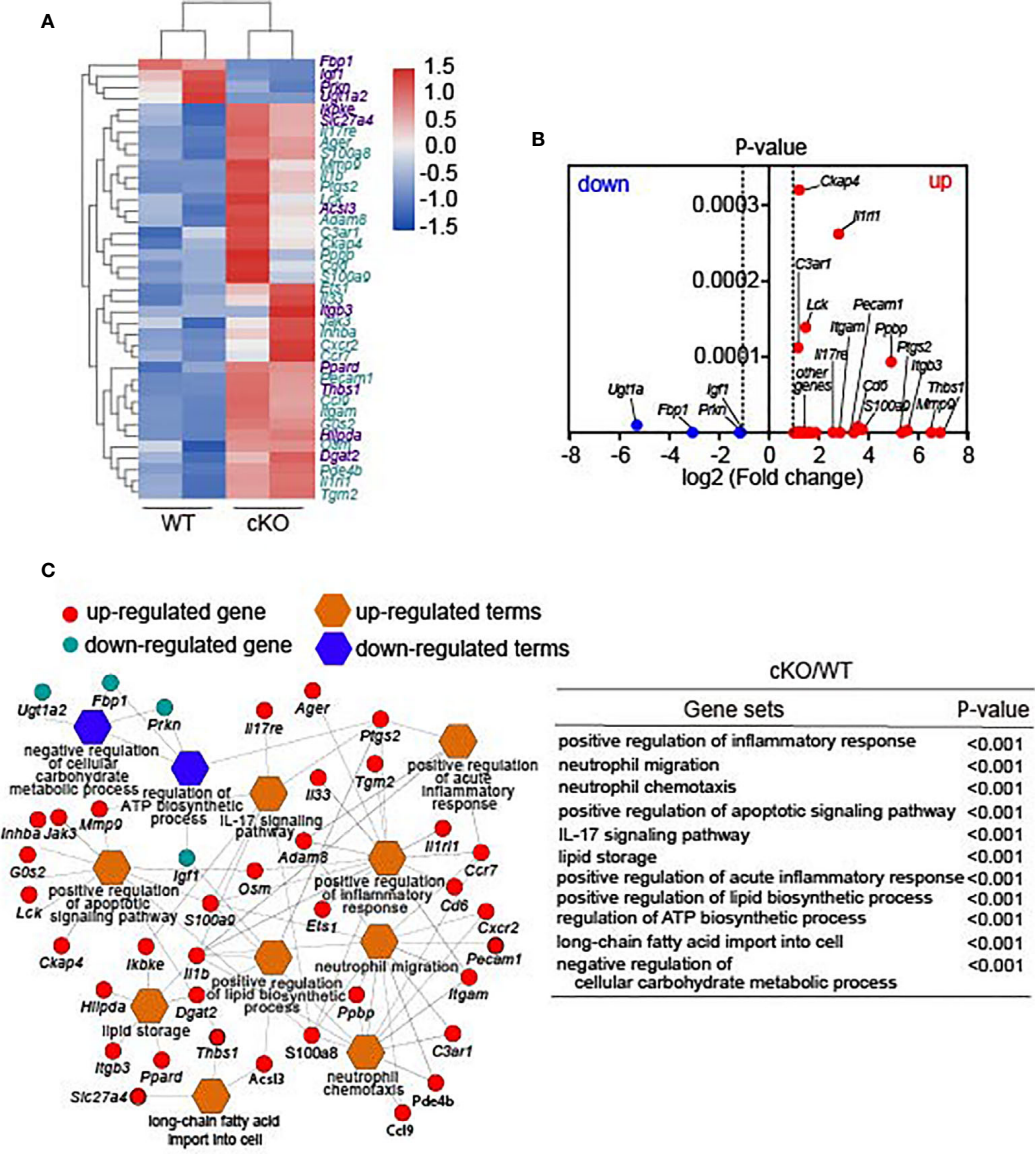
Lkb1 regulates critical immune and metabolic gene expression in AMs. **(A)** Heat map showing differentially expressed genes between AMs from *Lkb1* ^f/f^ and *Cd11c* ^Cre^ *Lkb1* ^f/f^ mice; genes labeled purple are related to metabolism, and genes labeled blue are related to immune function. **(B)** Volcano plot showing differentially expressed genes in Lkb1-deficient AMs compared to AMs from *Lkb1* ^f/f^ mice. **(C)** Network displaying the related genes involved in the pathways enriched in Lkb1-deficient AMs. The list (right) of gene sets and corresponding *P*-values are shown. CD45^+^ CD11c^+^ SiglecF^+^ AMs were sorted from the lungs of at least five to 10 mice in each sample with a FACSAria III (BD Biosciences). A *P*-value < 0.05 was considered statistically significant.

To explore the potential molecular mechanisms of Lkb1 in regulating the homeostasis and functions of AMs, we further analyzed the transcriptome sequencing of AMs sorted from *Cd11c* ^Cre^ *Lkb1* ^f/f^ and *Lkb1* ^f/f^ mice. Remarkably, there were 508 transcripts with a ≥2- or ≤-2-fold change in Lkb1-deficient AMs. Expression of a wide variety of genes critically involved in immune function was increased in Lkb1-deficient AMs, including those encoding secreted cytokines and chemokines (*Il1b*, *Il33*, *Cc19*), their related receptors (*Il1rl1*, *Il17re*, *C3ar1*) associated with immune activation, factors in the acute inflammatory response (*Ptgs2*) (26), and molecular and chemokine receptors in neutrophil migration and chemotaxis (*Itgb3*, *Itgam*, *Pecam1*, *Ccxr2*, *Ccr7*) (27). The increased expression of inflammatory mediators in Lkb1-deficient AMs might contribute to the more severe pathology in *Cd11c* ^Cre^ *Lkb1* ^f/f^ mice during *S. aureus* infection and allergic inflammation. Four genes were observed to have significantly reduced expression in Lkb1-deficient AMs, including *Igf-1*, *Fbp1*, *Prkn* and *Ugtla2*. Igf-1 enhances phagocytosis and bacterial killing in AMs (28, 29), which could explain the impaired bacterial scavenging capacity in AMs from *Cd11c* ^Cre^ *Lkb1* ^f/f^ mice during *S. aureus* infection. The *Ugtla2* gene regulates glycogen/glucose level and promotes the storage of glycogen (30), and the *Fbp1* gene encodes fructose-1,6-bisphosphatase 1, which inhibits glycolysis and tumor growth (31). Pathogenic variants in *Prkn* led to mitochondrial autophagy (32). In addition, some genes critically participating in lipid metabolism exhibited increased expression, such as fatty acid synthesis (*Dgat2*) (33) and transport (*Slc27a4*) and lipid synthesis, storage and β-oxidation (*Acsl3*, *Dgat2*, *Hilpda*) (34). *Inhba* and *Osm* genes, which inhibit cell proliferation, and the *G0s2* gene, whose function is promoting apoptosis, were increased in Lkb1-deficient AMs (**Figures 5A, B** **)** (35). Furthermore, GO (Gene Ontology) enrichment and pathway analysis revealed that positive regulation of the apoptotic signaling pathway was upregulated (**Figure 5C**, **Table S1**), which could explain the higher apoptosis proportion in Lkb1-deficient AMs. In addition, some pathways related to immune responses and metabolism were also enriched in Lkb1-deficient AMs (**Figure 5C**). Immune response-related pathways, including positive regulation of the (acute) inflammatory response and the IL-17 signaling pathway, were upregulated in Lkb1-deficient AMs, confirming the crucial function of Lkb1 in the suppression of the inflammatory response. Metabolism-related pathways, including positive regulation of the lipid biosynthetic process pathway, long-chain fatty acid import into cells and lipid storage, were upregulated in Lkb1-deficient AMs. The pathway for regulating the ATP biosynthetic process was downregulated in Lkb1-deficient AMs, which could be the potential mechanism through AM reduction occurs. These results suggest that Lkb1 operates as a pivotal mediator of AM immune protective function and metabolic homeostasis in the lung.

## Discussion

In our study, we demonstrated that mice with CD11c^+^ cell-specific deletion of Lkb1 exhibited a dramatically decreased number and percentage of AMs. However, the CD11c^+^ cell-specific deletion of AMPKα1 did not cause any reduction in AMs, indicating that the function of Lkb1 in maintaining the homeostasis of AMs was independent of the classical AMPK signaling pathway. Previous studies have shown that proliferative self-renewal is essential to maintain the AM population (36). However, the molecular mechanisms underlying the self-renewal of terminally differentiated AMs are not well understood. In our study, we found that loss of Lkb1 impaired AM self-renewal, reflected by increased apoptosis and reduced proliferation. Therefore, Lkb1 acts as a key regulator of AM self-renewal and homeostasis.

AMs play a crucial role in the maintenance of lung homeostasis and innate immune responses to pathogens (37). AMs certainly contribute to the development of severe inflammation (38–40), but they also play an important role in limiting excessive inflammation caused by infection. In the absence of AMs, influenza virus infection led to reduced viral clearance and increased inflammation and pathology (41–43). Here, we found that *Cd11c* ^Cre^ *Lkb1* ^f/f^ mice exhibited defects in bacterial scavenging capacity and aggravated infection in response to *S. aureus* pneumonia. It is well known that DCs are an essential bridge between innate and adaptive immunity that induce a T cell-specific immune response. AMs can suppress the induction of an adaptive immune response *via* their effects on alveolar and interstitial DCs and T cells. Similarly, increasing evidence suggests that AMs also have an important role in regulating T cell differentiation and the immune response in the lung. Previous studies have demonstrated that AMs suppress immune responses by inhibiting DC-mediated T cell activation and TGF-β production (44–46). A recent study revealed that CARD9^S12N^ could turn AMs into IL-5-producing cells, facilitating the pathologic responses mediated by Th2 cells (47). However, the mechanisms that underlie AMs regulating the immune response remain incompletely understood.

Interestingly, *Cd11c* ^Cre^ *Lkb1* ^f/f^ mice manifested excessive accumulation of neutrophils and a significant reduction in eosinophils in response to *S. aureus* pneumonia and asthma, indicating that Lkb1 plays a dominant role in maintaining the balance between eosinophilic and neutrophilic mediators of lung inflammation. Additionally, as shown in transcriptome analysis, the IL-17 signaling pathway was significantly enhanced in Lkb1-deficient AMs, which might lead to neutrophil accumulation and more severe pathology in *S. aureus* infection in *Cd11c* ^Cre^ *Lkb1* ^f/f^ mice. Recent study also demonstrated that *Lysm* ^Cre^ *Lkb1* ^f/f^ mice manifested more severe lung inflammation during *Klebsiella* pneumonia. These result indicated that Lkb1 is essential for local host defense during *S. aureus* and *Klebsiella* pneumonia by maintaining adequate AM numbers in the lung (48). In our study, we found that in addition to preserving AM abundance, Lkb1 may also protect the host from immune pathology and tissue damage by inhibiting expression of genes involved in immune activation and inflammation. Compared to other tissue macrophages, the different signatures involved in lipid metabolism of AMs highlighted their important role in the maintenance of airway homeostasis (49). Lkb1-deficient AMs might experience metabolic stress due to the lack of expression of cellular metabolic program-related genes, such as *Ugtla2*, *Fbp1* and *Prkn*. Although Lkb1-AMPK signaling could be activated under cellular stress conditions, increasing evidence has demonstrated that Lkb1 regulates lipid oxidation and reactive oxygen species (ROS) production independent of AMPK (50, 51). Therefore, it is unclear whether the cellular stress of Lkb1-deficient AMs is dependent or independent of AMPK signaling. AMPK conditional knockout mice had no effect on the AM pool, indicating that Lkb1 maintains the abundance of AMs independent of its classic downstream AMPK.

In conclusion, our results revealed an important role for Lkb1 in the maintenance of self-renewal and immune homeostasis in AMs. Lkb1 has crucial roles in suppressing signaling pathways related to the inflammatory response, including the IL-17 signaling pathway and neutrophil migration and chemotaxis signaling pathways. Our findings extend the understanding of AM homeostasis and function and the lung immune regulatory mechanism, indicating that the Lkb1 pathway may represent a potential therapeutic target to intervene in pulmonary inflammatory diseases.

## Data Availability Statement

We have submitted the RNA-Seq datasets to Gene Expression Omnibus (GEO) and the accession number is GSE167349.

## Ethics Statement

The animal study was reviewed and approved by the Institutional Animal Care and Use Committee at the Institute of Hematology, Chinese Academy of Medical Sciences.

## Author Contributions

QW, CS, and XF designed the study. QW, SC, and TL performed the experiments and analyzed the data. QY, JL, YT, YM, and JC helped with some experiments. QW and CS wrote the paper. XF edited the paper. EJ and XF helped to obtain funding for the research. ZH, HH, MS, MH, EJ, and XF oversaw the project. All authors contributed to the article and approved the submitted version.

## Funding

This study was supported by grants from the National Natural Science Foundation of China (81670171 to EJ, 81870090 to XF), the Nonprofit Central Research Institute Fund of Chinese Academy of Medical Sciences (2018PT32034 to HL, 2019-RC-HL-013 to XF), the CAMS Innovation Fund for Medical Sciences (CIFMS, 2016-12M-1-003), the Tianjin Science Funds for Distinguished Young Scholars (17JCJQJC45800 to XF), and the Fundamental Research Funds for the Central Universities (3332019095 to CS).

## Conflict of Interest

The authors declare that the research was conducted in the absence of any commercial or financial relationships that could be construed as a potential conflict of interest.
