# Supplementary figures and images for: Critical Role of Lkb1 in the Maintenance of Alveolar Macrophage Self-Renewal and Immune Homeostasis
Source: Front Immunol. 2021 Apr 22;12:629281. doi: 10.3389/fimmu.2021.629281 (PMC8100336; doi:10.3389/fimmu.2021.629281)

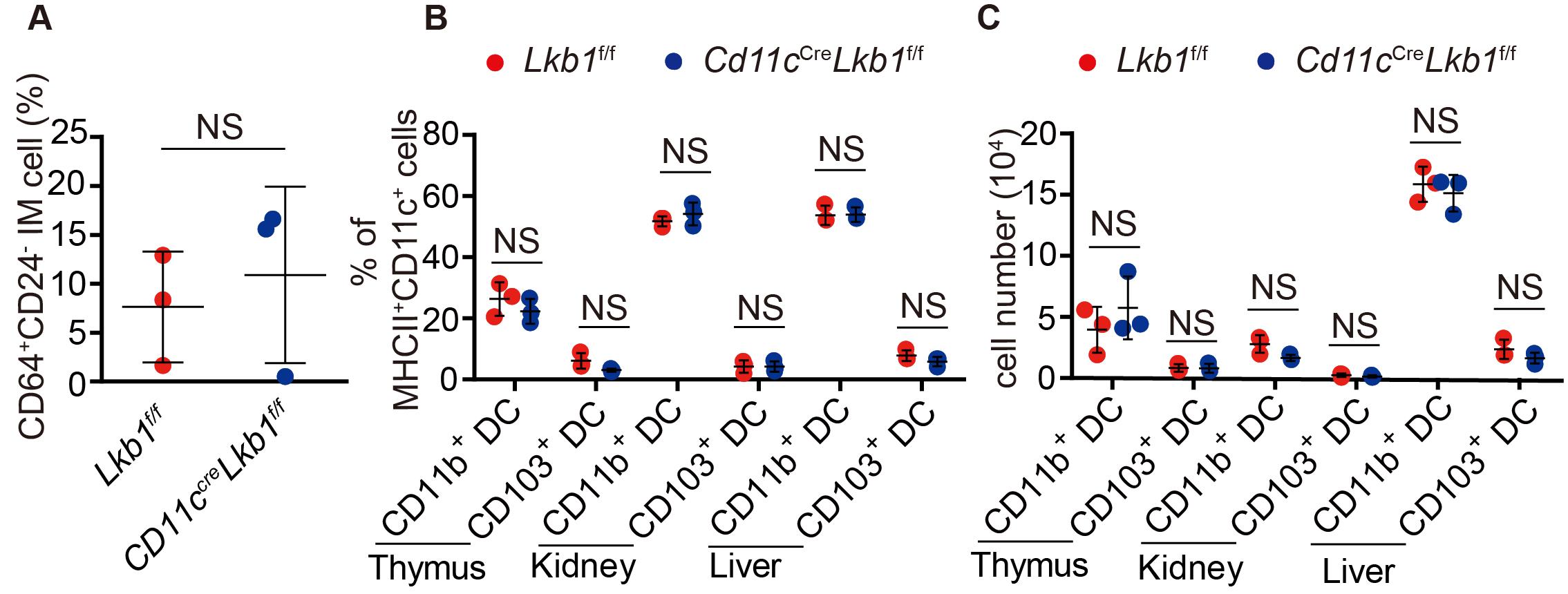

Supplement: Supplementary file 1 [file Image_1.jpg]

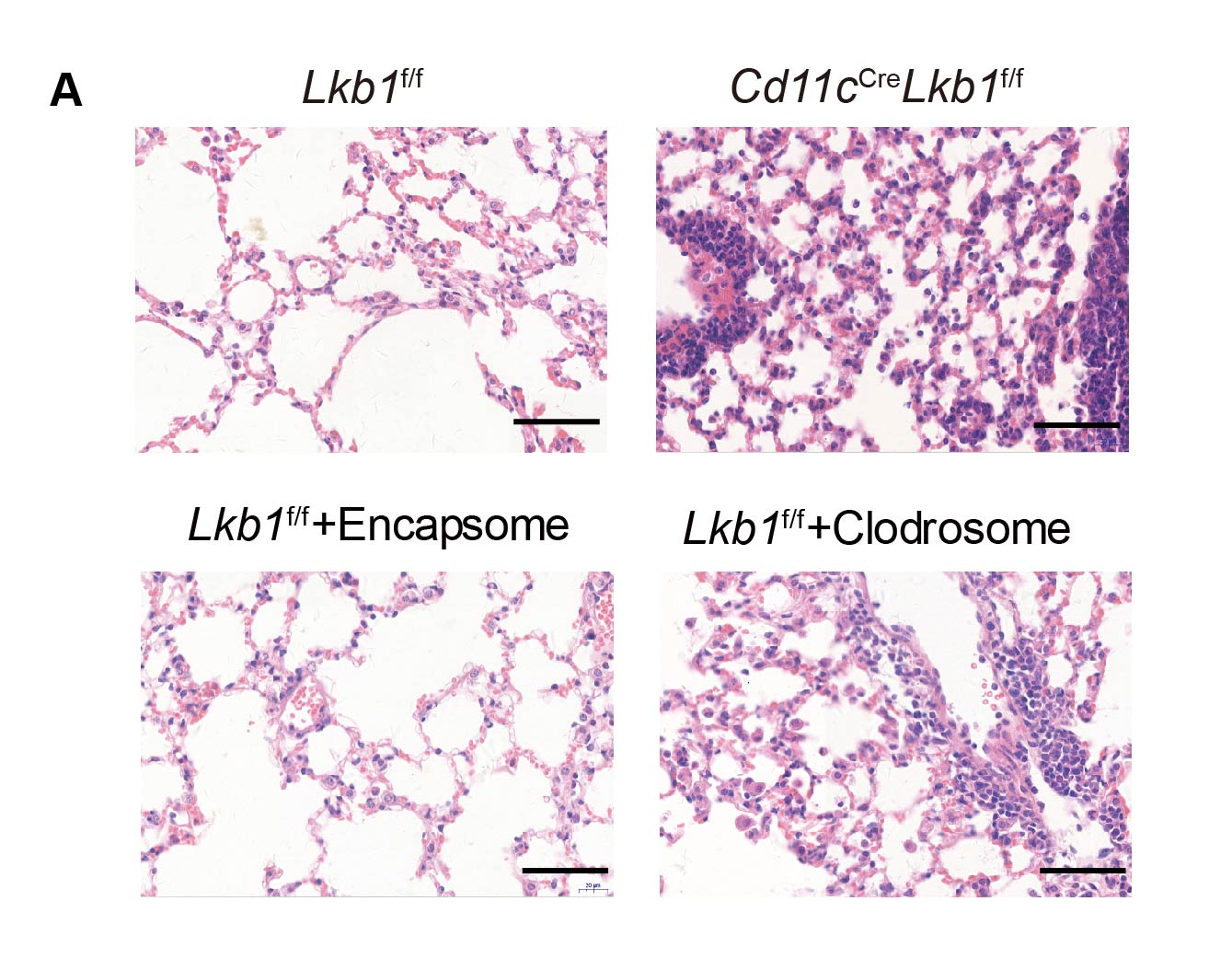

Supplement: Supplementary file 2 [file Image_2.jpg]

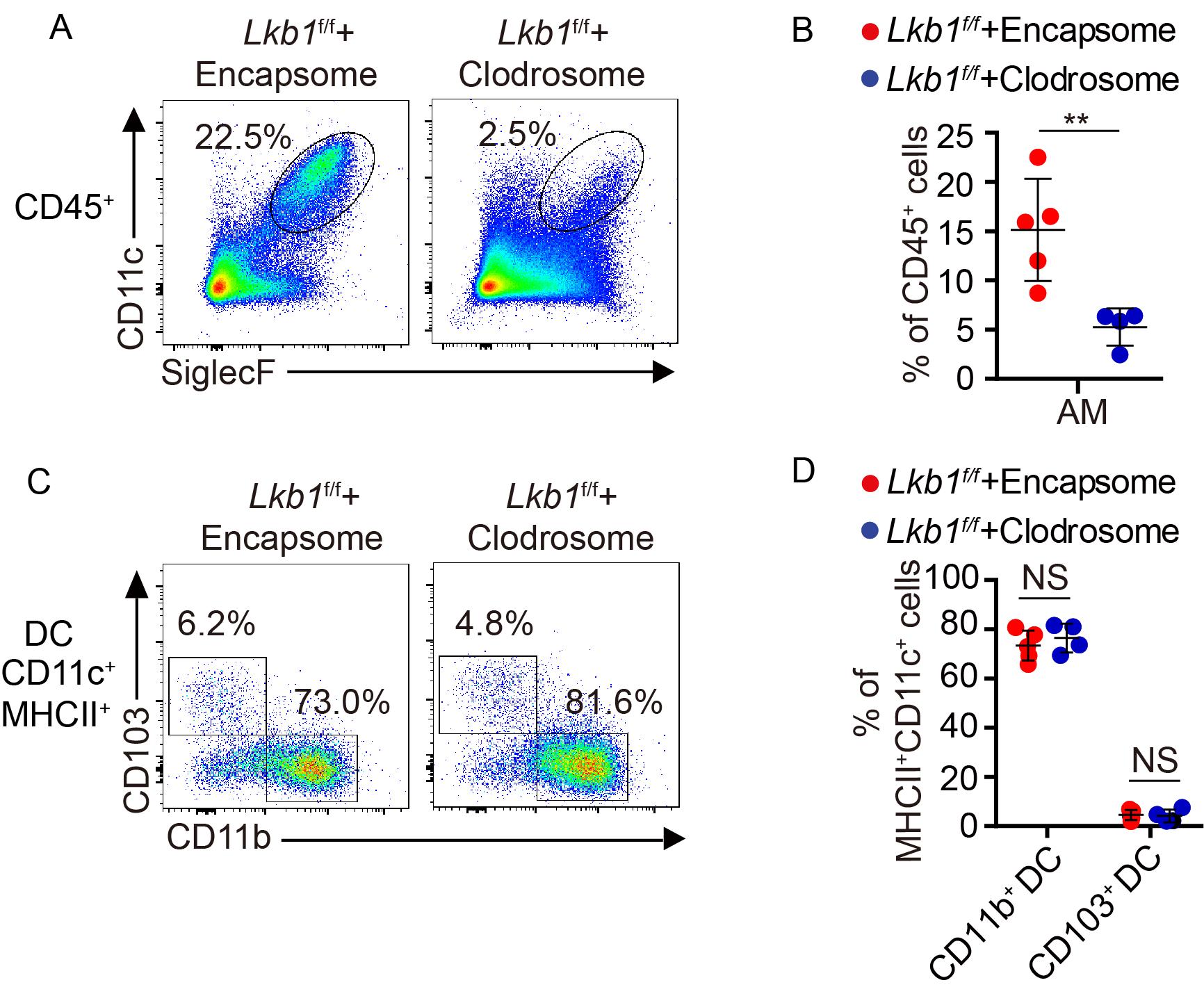

Supplement: Supplementary file 3 [file Image_3.jpg]

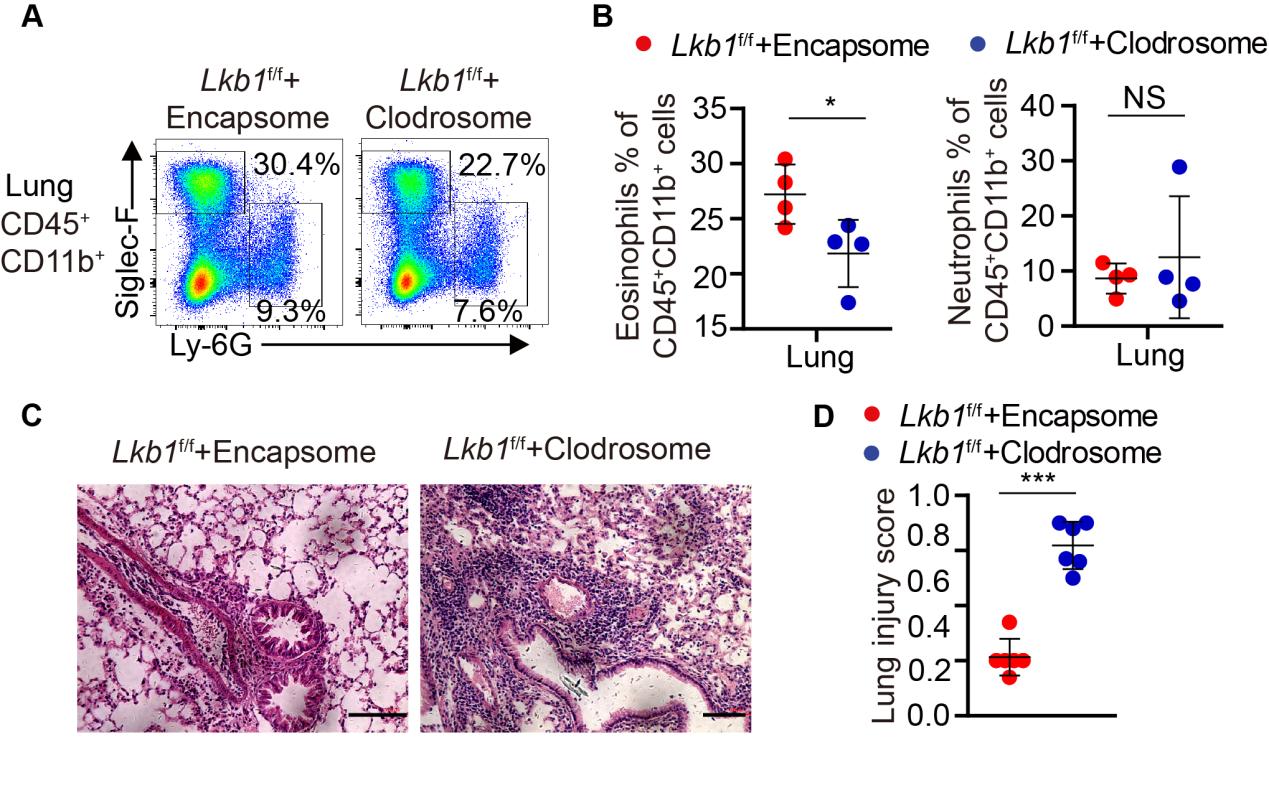

Supplement: Supplementary file 4 [file Image_4.jpg]

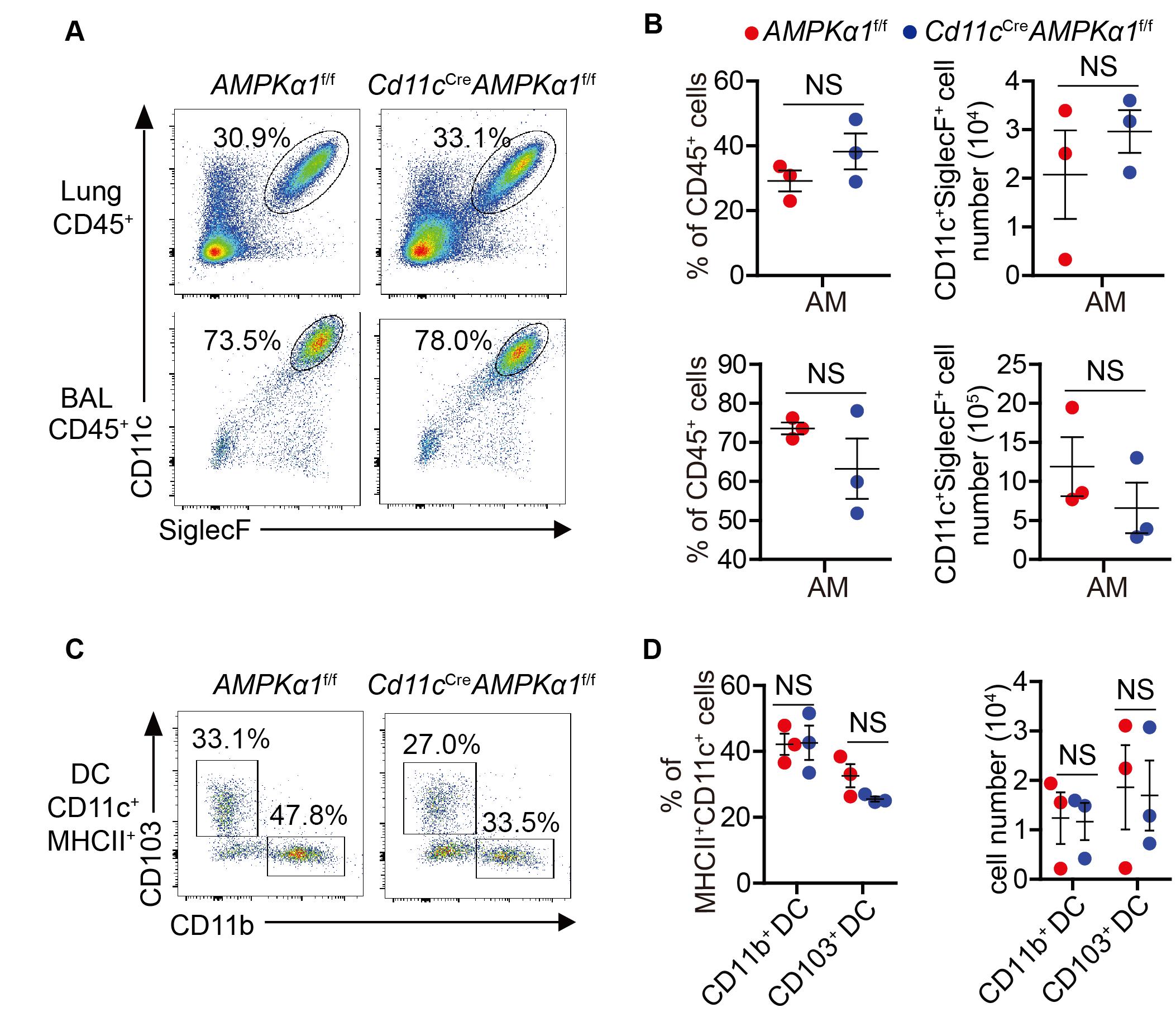

Supplement: Supplementary file 5 [file Image_5.jpg]
