# Supplementary material for: Critical Role of Lkb1 in the Maintenance of Alveolar Macrophage Self-Renewal and Immune Homeostasis
Source: Front Immunol. 2021 Apr 22;12:629281. doi: 10.3389/fimmu.2021.629281 (PMC8100336; doi:10.3389/fimmu.2021.629281)
Supplement: Supplementary file 6 [file Presentation_1.pdf]

Supplementary Figure legends

Figure S 1. Loss of Lkb1 did not affect the numbers of IMs or DCs in *Cd11c*$^{\mathbf{Cre}}$*Lkb1*$^{\mathbf{f/f}}$ mice.

(A) Quantification of the percentages of IMs from *Lkb1*$^{f/f}$ and *Cd11c*^{Cre}*Lkb1*$^{f/f}$ mice (n=3). (**B, C**) Quantification of the percentages (**B**) and absolute numbers (**C**) of CD103^{+} and CD11b^{+} DCs in thymus, kidney and liver from *Lkb1*$^{f/f}$ and *Cd11c*^{Cre}*Lkb1*$^{f/f}$ mice (n=3). (**D**) Flow cytometry analysis macrophages frequency among CD45^{+} leucocytes in BM, brain, kidney, liver, spleen and peritoneal cavity from *Lkb1*$^{f/f}$ and *Lysm*^{Cre}*Lkb1*$^{f/f}$ mice. (**E, F**) Quantification of the percentages (**E**) and absolute numbers (**F**) of macrophages in BM, brain, kidney, liver, spleen and peritoneal cavity from *Lkb1*$^{f/f}$ and *Lysm*^{Cre}*Lkb1*$^{f/f}$ mice (n=3-4). Each symbol (**A, B, C, E, F**) indicates an individual mouse; results are presented as mean ± S.D., NS, not significant ($P > 0.05$), by Student's *t*-test (**A, B, C, E, F**).

Figure S 2. Histopathological analysis of lung during *S. aureus* pneumonia

(A) Images show histopathological sections of lungs from *Lkb1*$^{f/f}$, *Cd11c*^{Cre}*Lkb1*$^{f/f}$ and *Lkb1*$^{f/f}$ mice intranasally administered Encapsome or Clodrosome, intranasally challenged with *S. aureus*, and stained with hematoxylin and eosin (scale bar, 20 μm).

Figure S 3. AMs were efficiently depleted in *Lkb1*$^{\mathbf{f/f}}$ mice after administrated with clodrosome intranasally.

(A) Flow cytometry analysis the frequency of AMs frequency among CD45^{+} leucocytes in lung from *Lkb1*$^{f/f}$ mice administrated with clodrosome or encapsome intranasally. (**B**) Quantification of the percentages of AMs in lung from *Lkb1*$^{f/f}$ mice administrated with clodrosome or encapsome intranasally (n=4-5). (**C**) Flow

cytometry analysis the frequency of $CD11b^+$ and $CD103^+$ DCs among $CD11c^+$ $MHCII^+$ DCs in lung from *Lkb1*$^{f/f}$ mice administrated with clodrosome or encapsome intranasally. (**D**) Quantification of the percentages of $CD11b^+$ and $CD103^+$ DCs in lung from *Lkb1*$^{f/f}$ mice administrated with clodrosome or encapsome intranasally (n=4-5). Each symbol (**B**, **D**) indicates an individual mouse; results are presented as mean ± S.D. NS, not significant ($P > 0.05$), $^{**}P < 0.01$, by Student's *t*-test (**B**, **D**). All data are representative of at least three independent experiments.

Figure S 4. AM depletion in *Lkb1*$^{f/f}$ mice developed severer pathology in asthma.

(A) Flow cytometry analysis of eosinophils ($CD45^+$ $CD11b^+$ Siglec-F^+) and neutrophils ($CD45^+$ $CD11b^+$ $Ly6G^+$) in BAL fluid and lungs from *Lkb1*$^{f/f}$ mice administrated with Clodrosome or Encapsome intranasally, challenged with HDM allergen intranasally. (**B**) Quantification of the percentages of eosinophils and neutrophils in BAL fluid and lungs from *Lkb1*$^{f/f}$ mice administrated with Clodrosome or Encapsome intranasally, challenged with HDM allergen intranasally (n=4-5). (**C**) Images show histopathological sections of lung from *Lkb1*$^{f/f}$ mice administrated with Clodrosome or Encapsome intranasally, challenged with HDM allergen intranasally, stained with hematoxylin and eosin (scale bar, 100 μm). (**D**) The lung injury score was evaluated blindly by two independent investigators (n=3-6). Each symbol (**B, D**) represents a mouse, the results are presented as mean ± S.D., NS, not significant ($P > 0.05$), $^{*}P < 0.05$, $^{**}P < 0.01$, $^{***}P < 0.001$ (by Student's *t*-test) (**B, D**). All data represent at least three independent experiments.

Figure S 5. Function of Lkb1 in AMs was independent of AMPK.

(**A**) Flow cytometry analysis of AMs frequency among $CD45^+$ leucocytes in lung and

BAL fluid from *AMPKα1*$^{f/f}$ and *CD11c*^{Cre}*AMPKα1*$^{f/f}$ mice. (**B**) Quantification of the percentages and absolute numbers of AMs in lung and BAL fluid from *AMPKα1*$^{f/f}$ and *CD11c*^{Cre}*AMPKα1*$^{f/f}$ mice (n=3). (**C**) Flow cytometry analysis of CD11b^{+} and CD103^{+} DCs frequency among CD11c^{+} MHCII^{+} DCs in lung from *AMPKα1*$^{f/f}$ and *CD11c*^{Cre}*AMPKα1*$^{f/f}$ mice. (**D**) Quantification of the percentages and absolute numbers of CD11b^{+} and CD103^{+} DCs in lung from *AMPKα1*$^{f/f}$ and *CD11c*^{Cre}*AMPKα1*$^{f/f}$ mice (n=3). Each symbol (**B, D**) indicates an individual mouse; results are presented as mean ± S.D., NS, not significant ($P > 0.05$), by Student's *t*-test (**B, D**). All data are representative of at least two independent experiments.
