# Supplementary material for: Critical Role of Lkb1 in the Maintenance of Alveolar Macrophage Self-Renewal and Immune Homeostasis
Source: Front Immunol. 2021 Apr 22;12:629281. doi: 10.3389/fimmu.2021.629281 (PMC8100336; doi:10.3389/fimmu.2021.629281)
Supplement: Supplementary file 7 [file Table_1.docx]

**Table S1.** The pathways and corresponding genes enriched in Lkb1-deficient AMs compared to AMs from *Lkb1*^f/f^ mice.

| **Go term** | **Genes** | ***P*-value** |
| --- | --- | --- |
| positive regulation of inflammatory response | *Adam8*, *Ager*, *Ccr7*, *Cd6*, *Ets1*, *Il1b*, *Il1rl1*,  *Il33*, *Itgam*, *Osm*, *Ptgs2*, *S100a8*, *S100a9*, *Tgm2* | 1.16E-20 |
| neutrophil migration | *Adam8*, *C3ar1*, *Ccl9*, *Ccr7*, *Cxcr2*, *Il1b*,  *Itgam*, *Pde4b*, *Pecam1*, *Ppbp*, *S100a8*, *S100a9* | 9.44E-18 |
| neutrophil chemotaxis | *C3ar1*, *Ccl9*, *Ccr7*, *Cxcr2*, *Il1b*, *Itgam*, *Pde4b*,  *Ppbp*, *S100a8*, *S100a9* | 8.08E-15 |
| positive regulation of  apoptotic signaling pathway | *Ckap4*, *G0s2*, *Inhba*, *Jak3*, *Lck*, *Mmp9*, *Osm*, *S100a8*, *S100a9*, *Thbs1* | 3.96E-12 |
| IL-17 signaling pathway | *Ikbke*, *Il17re*, *Il1b*, *Mmp9*, *Ptgs2*, *S100a8*,  *S100a9* | 2.55E-10 |
| lipid storage | *Dgat2*, *Hilpda*, *Ikbke*, *Il1b*, *Itgb3*, *Ppard* | 1.46E-08 |
| positive regulation of acute inflammatory  response | *Adam8*, *Ccr7*, *Il1b*, *Ptgs2* | 8.71E-07 |
| positive regulation of lipid biosynthetic  process | *Acsl3*, *Dgat2*, *Igf1*, *Il1b*, *Ptgs2* | 1.24E-06 |
| regulation of ATP  biosynthetic process | *Fbp1*, *Igf1*, *Prkn*, *Ptgs2* | 5.65E-06 |
| long-chain fatty acid  import into cell | *Acsl3*, *Slc27a4*, *Thbs1* | 6.05E-06 |
| negative regulation of cellular carbohydrate  metabolic process | *Fbp1*, *Prkn*, *Ugt1a2* | 0.000297 |
