# Supplementary material for: Critical Role of Lkb1 in the Maintenance of Alveolar Macrophage Self-Renewal and Immune Homeostasis
Source: Front Immunol. 2021 Apr 22;12:629281. doi: 10.3389/fimmu.2021.629281 (PMC8100336; doi:10.3389/fimmu.2021.629281)
Supplement: Supplementary file 8 [file Table_2.docx]

**Table S2. The antibodies we used for flow cytometry and their clones**.

| **Antibody** | **Clone** |
| --- | --- |
| [APC/Cyanine7 anti-mouse CD45](https://www.biolegend.com/en-us/products/apc-cyanine7-anti-mouse-cd45-antibody-2530) | [30-F11](https://www.biolegend.com/en-us/search-results?Clone=30-F11) |
| [PE/Cyanine7 anti-mouse CD45](https://www.biolegend.com/en-us/products/apc-cyanine7-anti-mouse-cd45-antibody-2530) | [30-F11](https://www.biolegend.com/en-us/search-results?Clone=30-F11) |
| [PerCP/Cyanine5.5](https://www.biolegend.com/en-us/products/percp-cyanine5-5-anti-mouse-cd45-antibody-4264) anti-mouse CD45 | [30-F11](https://www.biolegend.com/en-us/search-results?Clone=30-F11) |
| PE/Cyanine7 anti-mouse CD11c | [N418](https://www.biolegend.com/en-us/search-results?Clone=N418) |
| FITC anti-mouse CD11c | [N418](https://www.biolegend.com/en-us/search-results?Clone=N418) |
| PE anti-mouse CD170 (Siglec-F) | [S17007L](https://www.biolegend.com/en-us/search-results?Clone=S17007L) |
| APC anti-mouse CD170 (Siglec-F) | [S17007L](https://www.biolegend.com/en-us/search-results?Clone=S17007L) |
| APC anti-mouse I-A/I-E | [M5/114.15.2](https://www.biolegend.com/en-us/search-results?Clone=M5/114.15.2) |
| APC anti-mouse/human CD11b | [M1/70](https://www.biolegend.com/en-us/search-results?Clone=M1/70) |
| FITC anti-mouse/human CD11b | [M1/70](https://www.biolegend.com/en-us/search-results?Clone=M1/70) |
| [PerCP/Cyanine5.5](https://www.biolegend.com/en-us/products/percp-cyanine5-5-anti-mouse-cd45-antibody-4264) anti-mouse CD103 | [2E7](https://www.biolegend.com/en-us/search-results?Clone=2E7) |
| PE/Cyanine7 anti-mouse F4/80 | [BM8](https://www.biolegend.com/en-us/search-results?Clone=BM8) |
| FITC anti-mouse Ly6C | [HK1.4](https://www.biolegend.com/en-us/search-results?Clone=HK1.4) |
| APC/Cyanine7 anti-mouse Ly6C | [HK1.4](https://www.biolegend.com/en-us/search-results?Clone=HK1.4) |
| APC anti-mouse Ki-67 | [16A8](https://www.biolegend.com/en-us/search-results?Clone=16A8) |
| PE/Cyanine7 anti-mouse CD4 | [GK1.5](https://www.biolegend.com/en-us/search-results?Clone=GK1.5) |
| FITC anti-mouse/human CD44 | [IM7](https://www.biolegend.com/en-us/search-results?Clone=IM7) |
| APC anti-mouse IL-17A | [TC11-18H10.1](https://www.biolegend.com/en-us/search-results?Clone=TC11-18H10.1) |
| APC anti-mouse IL-4 | [11B11](https://www.biolegend.com/en-us/search-results?Clone=11B11) |
| PE anti-mouse/Rat Foxp3 | FJK-16s |
| APC/Cyanine7 anti-mouse CD45.1 | [A20](https://www.biolegend.com/en-us/search-results?Clone=A20) |
| FITC anti-mouse CD45.1 | [A20](https://www.biolegend.com/en-us/search-results?Clone=A20) |
| [PerCP/Cyanine5.5](https://www.biolegend.com/en-us/products/percp-cyanine5-5-anti-mouse-cd45-antibody-4264) anti-mouse CD45.2 | [104](https://www.biolegend.com/en-us/search-results?Clone=104) |
| PE anti-mouse CD45.2 | [104](https://www.biolegend.com/en-us/search-results?Clone=104) |
| APC/Cyanine7 anti-mouse Ly6G | [1A8](https://www.biolegend.com/en-us/search-results?Clone=1A8) |
| [PerCP/Cyanine5.5](https://www.biolegend.com/en-us/products/percp-cyanine5-5-anti-mouse-cd45-antibody-4264) anti-mouse Ly6G | [1A8](https://www.biolegend.com/en-us/search-results?Clone=1A8) |
| APC anti-mouse CD19 | [1D3/CD19](https://www.biolegend.com/en-us/search-results?Clone=1D3/CD19) |
